# Medicine quality in Somalia: Strengthening the pharmaceutical supply chain to protect patients

**DOI:** 10.1016/j.pmedr.2026.103626

**Published:** 2026-09-10

**Authors:** Mohamed Abdirahman Mohamed, Abdukadir Mohamed Hassan

**Affiliations:** a Department of Pharmacy, Dr. Sumait Hospital, SIMAD University, Mogadishu, Somalia.; b Department of Intensive Care Medicine, Dr. Sumait Hospital, SIMAD University, Mogadishu, Somalia.

**Keywords:** Medicine quality, Pharmaceutical supply chain, Patient safety, Pharmacovigilance, Somalia

## Abstract

**Objective:**

To highlight medicine quality as a patient-safety and public-health priority in Somalia and propose practical priorities for strengthening the pharmaceutical supply chain.

**Methods:**

This commentary draws on published literature and recent policy and regulatory developments concerning medicine quality, pharmaceutical governance, and supply-chain systems in Somalia and related settings.

**Results:**

Key vulnerabilities include limited regulatory and laboratory capacity, weaknesses in post-market surveillance, storage and distribution challenges, and insufficient integration of pharmacy professionals into medicine-quality monitoring. Recent legislative progress provides an opportunity to strengthen national regulation, but implementation, enforcement, financing, and technical capacity remain important.

**Conclusions:**

Somalia should strengthen risk-based regulatory inspection, practical post-market surveillance, supply-chain quality standards, product traceability and recall systems, and the formal role of pharmacists in medicine-quality monitoring and pharmacovigilance.

## 1 Commentary

Medicine quality in Somalia should be recognised as a patient-safety and public-health priority, not merely a procurement concern. Substandard medicines fail to meet quality specifications, while falsified medicines deliberately misrepresent their identity, composition, or source. Expired or improperly stored products may also compromise treatment. Ensuring that medicines are available, affordable, and of assured quality is fundamental to effective healthcare and universal health coverage. (World Health Organization, 2026; World Health Organization Regional Office for the Eastern Mediterranean, 2026)

Somalia has made important progress in pharmaceutical governance and supply systems, including a National Medicines Policy, an Essential Medicines List, a national supply chain, and a national medicines regulatory authority. Nevertheless, weaknesses across the pharmaceutical pathway—from selection and procurement to importation, storage, transportation, dispensing, and post-market surveillance—can undermine medicine quality. Evidence from Somalia and Somaliland has highlighted concerns related to regulatory capacity, laboratory limitations, porous borders, and the circulation of substandard and falsified medicines. (World Health Organization Regional Office for the Eastern Mediterranean, 2026; Ahmed and Brand, 2024) Infrastructure and workforce constraints further increase the importance of quality assurance across the supply chain.

The National Medicines Regulatory Authority has a central role in protecting patients through product registration, licensing, inspection, quality control, and market surveillance. Recent legislative progress provides an opportunity to strengthen this role. In January 2026, Somalia's House of the People approved the Somalia Medicines Bill. The regulatory authority stated that the Bill still required completion of constitutional procedures before becoming law. Its eventual impact will depend not only on enactment, but also on implementation, enforcement, sustainable financing, and technical capacity. (National Medicines Regulatory Authority, Somalia, 2026) Regulatory strengthening should therefore include risk-based inspection, appropriate reliance on trusted regulatory and World Health Organization prequalification mechanisms, and stronger systems for product traceability, reporting, and rapid recall.

Post-market surveillance also deserves greater attention. Rather than attempting to test every medicine, Somalia could adopt resource-sensitive approaches that combine case finding with risk-based sentinel surveillance at selected public and private facilities. Such approaches can identify priority quality risks while making efficient use of limited laboratory and regulatory resources. (Pisani et al., 2021) Storage, transportation, and distribution standards should also be strengthened, including temperature monitoring and adequate cold-chain capacity where required.

Pharmacists and other pharmacy professionals should be integrated into medicine-quality monitoring rather than viewed simply as dispensers. They can help maintain appropriate storage conditions, check product integrity and expiry, identify suspected poor-quality medicines, counsel patients, and report suspected adverse events or quality defects. Evidence from other settings demonstrates the potential contribution of community pharmacy professionals to controlling substandard and falsified medicines, while recent commentary has identified educational and regulatory gaps affecting pharmacy practice in Somalia. (Mekonnen et al., 2025; Sabrie, 2026)

Four priorities should therefore guide action in Somalia. First, strengthen regulatory capacity through risk-based inspection and sustainable financing. Second, establish practical post-market surveillance linked to laboratory testing, reporting, traceability, and rapid recall. Third, improve storage, transportation, and distribution standards across public and private supply chains. Fourth, formally integrate pharmacists into medicine-quality monitoring and pharmacovigilance through competency-based training and professional regulation.

Medicine quality is a shared system responsibility involving regulators, importers, suppliers, healthcare professionals, pharmacies, and policymakers. For Somalia, access to medicines should mean more than receiving a product; it should mean receiving a medicine of assured quality, safety, and efficacy.

## CRediT authorship contribution statement

**Mohamed Abdirahman Mohamed:** Conceptualization. **Abdukadir Mohamed Hassan:** Supervision.

## Consent for publication

Not applicable.

## Ethical approval and consent to participate

This commentary is based on published literature and did not involve human participants, animal subjects, or identifiable personal data. Therefore, ethical approval and informed consent were not required.

## Funding

The authors received institutional support from SIMAD University.

## Declaration of competing interest

The authors declare that they have no known competing financial interests or personal relationships that could have appeared to influence the work reported in this paper.

## Data Availability

No datasets were generated or analyzed during the preparation of this manuscript.
